# Promotions in Medical Department1*Weekly Bulletin*, No. 43. Chief Surgeon’s Office A. E. F.

**Published:** 1919

**Authors:** 


### PROMOTIONS IN MEDICAL DEPARTMENT[1]

1. On January 16th, 1919, the Chief Surgeon forwarded to G. H. Q, through Headquarters, S. O. S., a list of recommendations for promotion embracing 85 Lieut. Colonels to grade of Colonel, 282 Majors to grade of Lieut. Colonel, 832 Captains to grade of Major, and 2457 Lieutenants to grade of Captain. This list is a long one because there have been great and unfortunate delays in making promotions in the Medical Corps of the A.E.F., and it represents an accumulation to fill long existent vacancies. That this list does not exhaust the vacancies existing under the law is shown by the fact that if these promotions are made there will still remain the following vacancies: 241 in grade of Colonel, 293 in grade of Lieut. Colonel, 1151 in grade of Major, and 1323 in grade of Captain. It does not embrace all the medical officers in the A.E.F. who have earned their promotion and should be given it, but it does include those whose length of service in the A.E.F. and whose reports as to the character of service justify it.

2. It is certain that many of the medical officers serving with the British have not received the promotion to which the law and the character of their services have entitled them. They have failed to get their merited promotion, because in addition to the obstacles, delays and accidents which have been incidental to the history of promotions in the A.E.F. there was the added delay of mail communication with the B.E.F. and the great difficulty in getting from the 900 or more officers on duty with the British the reports of character of Service and Qualifications upon which the roster was based, which determined their promotion up to and including Major.

3. The necessity for making promotions in the Medical Corps of the A.E.F. has been especially urgent because most of the Reserve Corps Officers were commissioned in the lowest grade (Ist Lieut.), which was originally the only one, including men who had been fifteen or twenty years in practice. It was

1. *Weekly Bulletin*, No. 43. Chief Surgeon's Office A. E. F.

the intention of the Surgeon General to give these men prompt promotion as soon as their fitness for positions of increased responsibility was demonstrated, but the machinery for promotion has presented unexpected difficulties in the A.E.F., and therefore we find the proportion of Lieutenants to be about 60 per cent. instead of the 14 per cent. provided by law.

4. It seems appropriate in connection with this list to give a brief resume of the causes which have prevented a proper flow of promotions in the medical service of the A. E. F. and produced a stagnation which is greatly to be regretted.

5. Very few promotions were made during the first ten months of the existence of the American Expeditionary Forces. Those proposed by the Chief Surgeon were, as a rule, disapproved on the ground that a definite and methodical scheme of promotion, which would as nearly as possible do justice to all, should be presented before the Commander-in-Chief would be willing to make promotions, except in very exceptional cases. Such a scheme was finally worked out and presented to the Commanding General, S. O. S., on May 17, 1918, by whom it was forwarded May 19th with the following indorsement.

1st Ind.

"C. G., S. O. S., Am. E. F., France—19 May 1918—To the C. in C., G. H. Q., A. E. F.

1. Forwarded approved. Heretofore I have generally disapproved recommendations for promotion in the Medical Corps because they have come as isolated cases and presented no facts by which a reasonable judgment could be formed as to the relative merits of the particular case in comparison with the entire body of Medical Officers. As this paper presents a plan which appears to me to be comprehensive, legal and reasonable, I approve it and recommend that it be adopted as the basis for promotions of officers in this Corps serving with the A. E. F. in Europe.

E. J. Kernan,
Major General, N. A."

6. This plan was for the application of the principle of selection for the grades of Captain and Major by means of a roster on which each man took his place according to a roster number obtained by the addition of certain factors which were:

1. Age, which represents in a general way professional experience.
2. Military Service, which represents military experience.
3. Character of Service and Special Qualifications, which are given a numerical value in accordance with a special report made in each case by the immediate superior officer.

7. This scheme was approved in principle May 31st and finally approved by the Commander-in-Chief June 27th. The first list was forwarded on June 15th and five others in July. A number of other lists were forwarded in August. All lists after September 4 were held at G. H. Q. for action there under G. O. 78. Promotions of Lieutenants, however, were not cabled to Washington but sent by courier, and even in the case of those of higher grades the inevitable delays in the War Department made the process of getting through a promotion very slow. The entire summer passed without any promotions being received by Medical Reserve Officers except one group on June 6th. It was recently discovered that the list which was forwarded from this office on August 21st and sent by cable to the Adjutant General on September 2nd was apparently never received but lost in the cable office. This list contained the names of 54 Captains recommended for promotion to Major. A letter was recently written requesting that these nominations, which received the approval of the Commander-in-Chief more than four months ago, be now published as promotions of that date.

8. The Chief Surgeon was informed on September 4th by the Adjudant General, A. E. F., that a new War Department General Order on the subject of promotions (G. O. 78, W. D., August 22, 1918) was enroute from the United States. It was hoped that this order, which authorized promotions to be made by the Commander-in-Chief up to and including the grade of Colonel, subject to confirmation by the War Department, would greatly simplify and expedite promotions. This expectation was, however, disappointed because the opinion was advanced that in order to determine the question of whether vacancies existed an approved table of organization was necessary. It was then pointed out by the Chief Surgeon that as the law provided that there should be a certain proportion of medical officers in each grade, the number of vacancies in each grade could be readily determined by applying the proportions to the total number of medical officers in the A. E. F. The opinion was then expressed by the Judge Advocate's Office that the Surgeon General should be a party to this arrangement in order that the legal proportions in each grade should not be exceeded for the Medical Department with the other forces. The question of the applicability of G. O. 78 to the Medical Department was finally referred to the War Department by cablegram of the C-in-C dated October 11th, which was replied to by the Chief of Staff on October 19th stating that it *did not apply* to officers in the Medical Department. It is understood that the negative in this cable was an error in coding. When the matter was again presented by the C-in-C on October 28th for reconsideration, he was informed by cable of November 5th that his request for authority to promote medical officers was approved. The Chief Surgeon was informed of this decision on November 7th, but four days later the armistice was signed and stopped all temporary promotions. During this short period, however, 672 Medical, 32 Sanitary, and 53 Veterinary officers were promoted.

